# Rapid Assessment of Ocular Toxicity from Environmental Contaminants Based on Visually Mediated Zebrafish Behavior Studies

**DOI:** 10.3390/toxics11080706

**Published:** 2023-08-16

**Authors:** Jia Yi, Yilei Ma, Jiahui Ma, Haiyang Yu, Kun Zhang, Libo Jin, Qinsi Yang, Da Sun, Dejun Wu

**Affiliations:** 1 Institute of Life Science & Biomedical Collaborative Innovation Center of Zhejiang Province, Wenzhou University, Wenzhou 325035, China; 2 Bioengineering College of Chongqing University, Chongqing 400030, China; 3 National and Local Joint Engineering Research Center for Ecological Treatment Technology of Urban Water Pollution, College of Life and Environmental Science, Wenzhou University, Wenzhou 325035, China; 20160121@wzu.edu.cn; 4 Wenzhou Institute, University of Chinese Academy of Sciences, Wenzhou 325000, China; yangqs@wiucas.ac.cn; 5 Emergency Department, Quzhou People’s Hospital, Quzhou 324000, China

**Keywords:** rapid assessment, Zebrafish, environmental contaminants, ocular toxicity, behavior

## Abstract

The presence of contaminants in the environment has increased in recent years, and studies have demonstrated that these contaminants have the ability to penetrate the blood–retinal barrier and directly affect the visual systems of organisms. Zebrafish are recognized as an ideal model for human eye diseases due to their anatomical and functional similarities to the human eye, making them an efficient and versatile organism for studying ocular toxicity caused by environmental contaminants in the field of environmental toxicology. Meanwhile, zebrafish exhibit a diverse repertoire of visually mediated behaviors, and their visual system undergoes complex changes in behavioral responses when exposed to environmental contaminants, enabling rapid assessment of the ocular toxicity induced by such pollutants. Therefore, this review aimed to highlight the effectiveness of zebrafish as a model for examining the effects of environmental contaminants on ocular development. Special attention is given to the visually mediated behavior of zebrafish, which allows for a rapid assessment of ocular toxicity resulting from exposure to environmental contaminants. Additionally, the potential mechanisms by which environmental contaminants may induce ocular toxicity are briefly outlined.

## 1. Introduction

**Figure 1:**
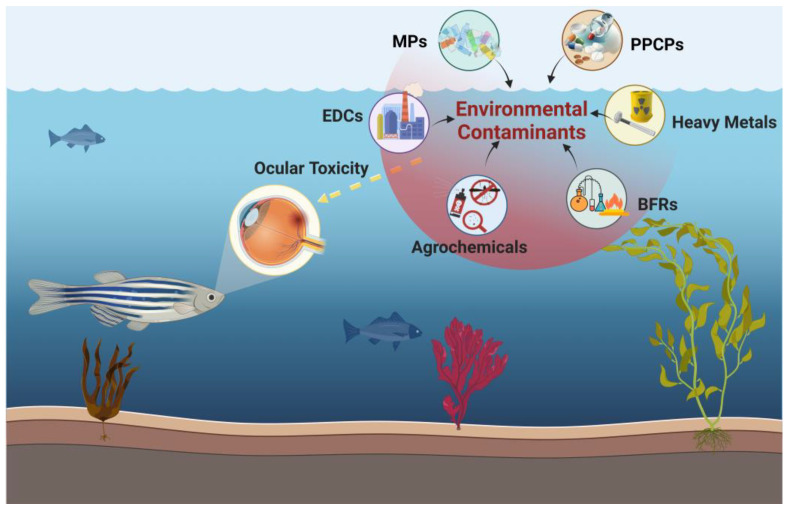
Assessing the ocular toxicity of environmental contaminants through visually mediated zebrafish behavioral studies. (Brominated flame retardants, BFRs; Microplastics, MPs; Pharmaceutical and personal care products, PPCPs; Endocrine disrupting chemicals, EDCs).

As a superficial organ, the eye is frequently directly exposed to the surrounding environment, leaving it susceptible to direct or indirect harm from hazardous elements in the environment that can cause eye injury [1,2]. With an estimated 295 million people worldwide suffering from moderate and severe visual impairment as of 2020, including approximately 43.3 million who are even blind [3], environmental contaminants are gaining attention as an important direct or indirect causative factor [4,5]. Meanwhile, several epidemiological studies suggest that environmental pollutants may be associated with congenital eye malformations [6]. These include exposure to environmental chemical pollutants during pregnancy or during the early stages of newborn life, which may cause vision problems in newborns through the placenta or breast milk [7,8,9,10]. As shown in Figure 1, environmental contaminants that have been shown to act on the visual system, which include endocrine disrupting chemicals (EDCs), brominated flame retardants (BFRs), pharmaceutical and personal care products (PPCPs), microplastics (MPs), agrochemicals, and heavy metals, which adversely impact embryonic development and the visual performance of the neural retina of organism [11], causing multiple visual system defects at different levels of biological tissues (Figure 1) (e.g., genes, proteins, organology, and physiology) [12,13,14]. Moreover, water-insoluble contaminants are commonly investigated through the utilization of organic solvents, among which are dimethyl sulfoxide, methanol, and acetone, serving as co-solvents [15,16,17]. In the face of such a severe threat from environmental contaminants, there is an urgent need for a scientifically rational animal model to be developed for a rapid and accurate evaluation of the ocular toxicity of environmental contaminants on organisms and to study their mechanisms.

The zebrafish (*Danio rerio*), a typical biological model in ecotoxicology research, has a fully sequenced genome, with the majority of zebrafish genes shared with humans [18]. Furthermore, 84% of known human disease-related genes have zebrafish counterparts, and zebrafish exhibit a wide range of morphological and biochemical similarities with humans [19,20]. Since visual development and functional databases for the zebrafish are now accessible, they have become as an essential model in hard-boned fish research for toxicological investigations [21].

Similar to mammals, zebrafish also possess a variety of social behaviors, including social interaction, anxiety, learning, memory, and defense mechanisms [22,23,24]. Foraging and predator avoidance are both closely related to vision, which is the most fundamental sensory system in zebrafish. In addition, environmental contaminants can accumulate in the eyes of an organism, altering the expression of essential visual proteins, disrupting retinal structure, and impairing the function of the visual system [25,26,27], so behavioral endpoints can be utilized to rapidly investigate the effects of pollutants on the visual system. Such behavioral studies in living organisms can provide a more comprehensive understanding of the effects of contaminants [28].

Furthermore, the advantage of zebrafish models in ophthalmic research is derived from the similarities between human and zebrafish embryo eye development architecture, as well as the remarkable adaptability of zebrafish to experimental and genetic modification [29]. In comparison to mammals, which have long cycles, large economic costs, and complex operations, zebrafish exhibit high fecundity fertilization capacity in vitro, a quick embryonic development process, a small size, and plentiful embryonic resources, making them excellent for high-throughput screening [30].

This review comprehensively introduces the ocular anatomy and function of zebrafish, summarizes its methods for rapidly evaluating the ocular toxicity of environmental contaminants, and describes the underlying mechanisms responsible for such toxicity. Finally, the challenges and prospective development trends in utilizing the zebrafish model to assess the ocular toxicity of environmental contaminants are further discussed.

## 2. The Eye Structure of Zebrafish

**Figure 2:**
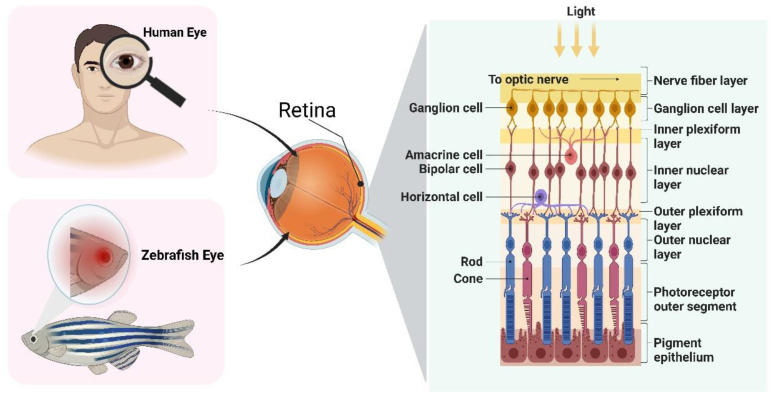
Structural similarities between the human and zebrafish retina.

The zebrafish has emerged as a promising model for studying human eye disorders due to its remarkable similarities in anatomy, function, tissue composition, circuitry, and biochemistry with other vertebrates [31]. The highly conserved nature of zebrafish eyes among vertebrates has facilitated the successful modeling of numerous eye disorders [32], including cataracts, glaucoma, diabetic retinopathy, and age-related macular degeneration [16,33]. Zebrafish retinas exhibit the typical morphology found in vertebrate retinas, resembling adult retinas, and share both anatomical and functional similarities with human retinas [29,34]. While the macula is a cone-shaped, dense region responsible for central high-resolution color vision in the human retina [35], recent discoveries have identified a comparable macular area in the retinas of zebrafish larvae, which plays a crucial role in extremely sensitive vision, akin to the human macula [36,37]. Furthermore, the arrangement of neural networks and gene expression patterns in zebrafish photoreceptors closely resemble those observed in humans [38]. Consequently, the zebrafish has emerged as the preferred model for investigating human retinal diseases (Figure 2).

The retina of the zebrafish consists of three layers: the outer nuclear layer (ONL), the inner nuclear layer (INL), and the ganglion cell layer (GCL), which are separated by synapse-rich plexiform layers (outer plexiform layer, OPL; inner plexiform layer, IPL) [35]. There are the same retinal neurons in zebrafish as in humans, including retinal ganglion cells (RGC), bipolar cells, horizontal cells, and amacrine cells, as well as the same glial cells, including Müller cells, astrocytes, and microglia [39]. The retina is covered by Müller glial cells, which serve as scaffolds for neurons, interact with all retinal neuronal bodies and processes, and contribute to the regulation of neurotransmitters as well as gas and ion homeostasis [40,41]. The ONL consists of one rod type and four morphologically and spectrally distinct cone subtypes, namely short single cones (ultraviolet (UV)-sensitive), long single cones (blue-sensitive), double cone accessory members (green-sensitive), and double cone principle members (red-sensitive) [42,43]. The INL is composed of interneurons (bipolar cells, horizontal cells, and amacrine cells) and Müller glia [36]. The plexus layer lies between the nuclear layers and serves as the primary site of synaptic connections between retinal neurons. The OPL contains photoreceptors, bipolar cells, and horizontal cells, while the IPL consists of bipolar cells, amacrine cells, and ganglion cells [36].

ONL is responsible for the initial step in light signal reception and conversion into electrical signals [44]. When ambient light enters the eye, it reaches the retina through the refractive medium of the bipolar cells and photoreceptors, with the latter playing a crucial role in this process. Visual signals from photoreceptors, including cones and rods, are transferred from the retina to ganglion cells in the GCL and subsequently relayed to the brain [45]. Additionally, the retinal pigment epithelium (RPE), which is a layer of pigment cells located outside the neurosensory retina, supports the retinal vision cells and serves as a critical interface between photoreceptors and the choroid in the vertebrate eye [11,46]. Extensive molecular interactions occur between the photoreceptors in the neural retina and the RPE [40].

Zebrafish provide reliable endpoints for the study of retinal injury and the investigation of developmental toxicity in the ocular system as an excellent vertebrate model. One prominent method utilized in both rodent and zebrafish retina research is optical coherence tomography retinal imaging [47]. This non-invasive interference technique provides cross-sectional and facial images of the rodent and fish retina, enabling precise identification of minimal lesions and significant changes in retinal structure, thus achieving high-resolution retinal imaging [35,48]. Yet, it is worthwhile to emphasize that a variety of behavioral paradigms can be utilized to quickly, intuitively, and directly identify impairments in zebrafish visual function. Visually mediated behavioral changes have been widely utilized to evaluate the ocular toxicity of pollutants in the early stages of zebrafish life [49]. In visual research, mice and rats are commonly used as primary animal models, whose retinas are mainly composed of rod cells, resulting in color vision defects or loss, relatively low visual acuity, and more reliance on olfactory, tactile, and auditory cues in bright environments [35]. In contrast, the zebrafish serves as a valuable animal model for studying visually mediated behaviors, offering notable advantages. Firstly, zebrafish have a cone-dominated vision similar to humans, unlike mice, which have rod-dominated vision. Zebrafish possess cone photoreceptors that are sensitive to UV, blue, green, and red light, resulting in excellent color vision and visual acuity [35,36]. Secondly, zebrafish exhibit strong reproductive capabilities, with externally developing embryos that are optically transparent, facilitating easy observation. Additionally, zebrafish have relatively large eyes in proportion to their body size, allowing for manipulation of the eye bud during early embryonic development [39,50]. Thirdly, the visual system of zebrafish develops rapidly, with eye development completed by 72 h post-fertilization (hpf), enabling the direct observation of morphological defects within a short timeframe. Functional assessments can be conducted after 120 hpf, enabling faster identification of issues related to mature visual acuity compared to mice, which typically take around 15–20 days [51]. Fourthly, the small size of zebrafish makes them well-suited for behavioral tests in multiwell plates, and their behavior can be effortlessly monitored using automated video tracking systems [26]. Lastly, both adult zebrafish and larvae exhibit easily analyzable visual behaviors without the need for training, and there exist various robust behavioral paradigms for rapidly assessing the visual function of zebrafish and detecting visual impairments [35,40].

## 3. Quick Approaches for Assessing Ocular Toxicity

Behavior integrates the responses of animals to internal and external stimuli and can be used as an indicator of the effects of environmental pollutants on animals at molecular, biochemical, and physiological levels [52]. Such behavioral disorders arise from changes in neuronal structure and/or physiology. Particularly, zebrafish behavior exhibits high sensitivity to environmental pollutants, making it a particularly effective model for assessing the toxic effects of these pollutants [52]. In recent times, Zebrafish behavior has been quantitatively assessed in a growing number of studies to evaluate the toxicity endpoints of pollutants in zebrafish [53,54].

**Figure 3:**
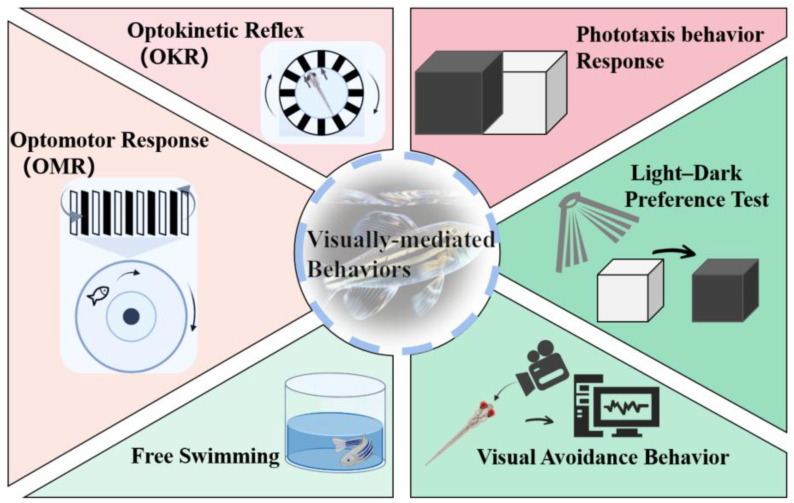
Visually mediated behavioral paradigm in zebrafish.

Zebrafish possess a visual processing circuitry that comprises the neural retina, optic nerve, optic tectum, and extraocular muscles. Their behavior heavily relies on a mature retina, which enables stable high-resolution imaging and eye movement reflexes that respond to fast-moving visual stimuli [51]. The activity of innate visual reflexes supports a variety of visually mediated behaviors and effectively assesses the visual function of zebrafish [11]. Several commonly used methods for studying zebrafish behavior include the optokinetic reflex (OKR), optomotor response (OMR), phototaxis, light–dark preference test, free-swimming activity, and visual avoidance behavior analysis (Figure 3) [11,35,55]. These behavioral responses have been extensively utilized in toxicology studies to identify different types of visual cues driving behavior, genes involved in vision and visual impairments, and the specific neurons and circuits responsible for perceiving visual stimuli and eliciting behavioral responses, often utilizing in vivo functional imaging techniques [32].

### 3.1. OKR

OKR is a convenient method for evaluating visual function in awake animals, as it provides visual stimulation and relies on the functional retina without the need for training [56]. The test typically involves immobilizing zebrafish in a transparent tank and stimulating them with gradually increasing or decreasing circular or vertical stripes, allowing only their eyes to move as they track the rotating stripes, followed by observing their eye movements [51,57]. This behavior matures between 73 and 80 hpf and persists throughout adulthood [58]. During OKR testing, fish with expected visual function deficiencies are anticipated to exhibit a reduced frequency of eye scanning or abnormal eye movements [59,60]. However, there are variations in the experimental protocol for conducting the OKR test in practice. These differences include the growth stage of the zebrafish, fixation methods, rotation speed of the stripe pattern, and the parameters used for behavioral evaluation of the experimental zebrafish, as indicated in Table 1.

OKR is also frequently used in assessing visual function in larvae, and it has been employed in studies investigating the effects of environmental contaminants on zebrafish [16]. These investigations have revealed that zebrafish larvae exposed to certain polybrominated diphenyl ether (PBDE) congeners, such as 2,2′,4,4′,5-pentabromodiphenyl ether (BDE99), exhibited decreased saccadic responses and responses to blue and green light stimuli in OKR tests assessing visual behavior [16]. Similarly, exposure to polycyclic aromatic hydrocarbons (PAHs) present in crude oil led to diminished eye movements [61], while exposure to the plasticizer bisphenol S (BPS) significantly impaired eye-tracking ability during visual behavior OKR testing [56]. Furthermore, exposure to triphenyl phosphate (TPhP) and triclocarban (TCC) was found to reduce the OKR response, with TPhP demonstrating a dose-dependent decrease [44,62]. In general, exposure to these environmental contaminants may have detrimental effects on the visual function of zebrafish. However, it is important to note that exposure to certain environmental contaminants can also cause zebrafish to exhibit a positive OKR. For instance, exposure to the commercial PBDE mixture, DE-71, significantly increased visual–motor response [63].

**Table 1:** Comparative analysis of variations in OKR implementation process.

Compounds	Stage	Apparatus	Stripe Pattern	Behavior Evaluation Parameters	Ref.
BDE-99	120 hpf	35 mm Petri dish containing 3% methylcellulose	Rotational black and white stripes (10 rpm)	Saccadic movements per minute	[16]
Crude Oil	96 hpf	35 mm Petri dish Containing 3% methylcellulose	Rotating black and white vertical stripes (6 rpm)		[61]
DE-71	15 dpf	35 mm Petri dish containing 6% methylcellulose	A drum lined with 18° vertical black and white stripes. (8.1 rpm)		[63]
BPS	120 days post-fertilization(dpf)	A specially constructed cylindrical water-filled glass chamber	Rotating drum with black and white stripes (Unstated speed)	Slow phase gain	[56]
TPhP	144 hpf	35 mm Petri dish containing 6% methylcellulose	Sine-wave grating (with a constant angular velocity of 7.5 per second and a spatial frequency of 0.6 cycles per degree)	Ratio of eye velocity to stimulus velocity	[44]
TCC	144 hpf	Fixed in a glass slide with 1.6% agarose solution	Moving vertical black-white, blue-white, green-white, and red-white stripes (12 cycles/360°, 30°/s)		[62]

### 3.2. OMR

The OMR is a rapid, straightforward, and efficient method for assessing visual function in zebrafish [32], which involves visual stimulation of the fish with moving stripe patterns presented alternately from below or beside the fish, consisting of vertical stripes of different colors, such as black and white, gray with different contrasts, or other different colors [32,64]. The OMR is an innate visuomotor reflex behavior in which fish swim in the same direction as high-contrast visual stimuli [65]. These behaviors may be displayed both while swimming freely or while immobilized with the head of zebrafish larvae embedded in agarose while allowing the trunk and tail to move [32].

Scoring the response of larvae is possible through video recordings of their swimming behavior, typically with fixed larvae, or by recording the distance moved relative to the direction of the stimulus [32]. The latter approach can be performed in a high-throughput manner using 384-well plates [32]. While OMR is similar to OKR, the stimulus in OMR drives the movement of the head and body rather than the eyes. These tests have different visual functional features, while OMR and OKR test results overlap. OMR emphasizes the visual motor ability of adult zebrafish, which depends on functional retinal projection [56]. Additionally, the design of OMR experiments may vary depending on the developmental stage of the zebrafish, the experimental equipment used, and the parameters for behavioral evaluation, as presented in Table 2.

Zebrafish larvae exposed to polychlorinated biphenyls, specifically PCB1254, exhibited abnormal behavior in OMR tests, with a significant reduction in the proportion of actively swimming fish compared with control larvae [15]. Similarly, male zebrafish exposed to BPS exhibited a significant reduction in their tracking ability during OMR testing [56]. However, no significant difference was observed in the OMR tests of zebrafish following acute exposure to the insecticides atrazine and diazauron, as all groups exhibited positive visuomotor responses [66].

**Table 2:** Comparative analysis of variations in OMR implementation process.

Compounds	Stage	Apparatus	Behavior Evaluation Parameters	Ref.
PCB1254	7 dpf	Transparent test tanks with black and white stripes are generated using custom-designed raster software.	The proportion of fish that positive swimming, opposite swimming, hesitant swimming, and immobility	[15]
BPS	120 dpf	A round transparent tank with an opaque column in the middle and a rotating drum with black and white stripes outside the tank. (Turn the drum clockwise for 1 min, then counterclockwise)	Concordance ratio (the quotient of male zebrafish stripe tracking time divided by the whole recording time)	[56]
Atrazine, diazauron	7 dpf	Petri dishes over an LCD monitor with moving animations in black and white stripes	Proportion of zebrafish that moved to the end of the dish following the stripes	[66]

### 3.3. Phototaxis Behavior Response

Phototaxis is an innate behavior in which zebrafish larvae exhibit a response to light, whereby changes in the sensitivity of their eyes to light stimuli decrease the perceived difference between light and dark areas [16,49]. The phototaxis behavioral response test serves as a rapid and straightforward screening tool used to assess eye defects induced by environmental contaminants [44,67,68]. This test capitalizes on the natural inclination of zebrafish to move toward the lighted chamber, enabling the assessment of the coordination between motor movements and sensory perception in their visual system. It is widely employed to identify recessive visual defects in model biological systems [16,35]. To prevent any discrepancies in individual larva movements, zebrafish larvae with normal swimming function are typically selected for investigations.

The phototaxis behavioral response test typically involves the utilization of a rectangular acrylic box with a sliding partition that separates compartments A and B. A specific number of zebrafish larvae are placed in darkened chamber B for a predetermined period. Once the larvae have acclimated to the dark environment, the partition is opened to expose chamber A to ambient light, while chamber B remains dark. The larvae in each chamber are counted, and the percentage of larvae that swim from the dark chamber to the light chamber after a designated time is used to assess their responsiveness to light. The test can be conducted using blue, green, and red light in the lighting room to evaluate the impact of color perception. To assess whether the larvae accidentally entered chamber A, a controlled experiment was performed in which the chamber remains darkened after the partition is raised [62]. The detailed experimental protocol is shown in Table 3.

Environmental contaminants have the potential to induce functional impairments in the eye or visual processing centers, resulting in damage to photoreceptors, retinal morphological changes, and cell apoptosis [46]. Photoreceptors and their accompanying opsins are particularly sensitive to exogenous substances among the numerous components of the visual system [16]. Several studies have demonstrated that zebrafish larvae are less responsive to light after being exposed to environmental contaminants [69].

The phototaxis behavior of zebrafish larvae was observed to be inhibited following a single exposure to TCC [62], BDE99 [16], phenanthrene polycyclic aromatic hydrocarbon (Phe) [46], TPhP [44], and the agricultural chemical boscalid [17]. Notably, zebrafish larvae exposed to TPhP exhibited a dose-dependent reduction in the phototaxis response [44]. Various wavelengths of light signals (short, medium, and long) were employed to assess the ability of zebrafish larval cone cells to perceive different wavelength spectra. It was found that exposure to BPS inhibited the phototaxis of zebrafish larvae across all wavelength bands (short, medium, and long) in order to test the ability of larval cone cells to perceive varying wavelength spectra [70]. In contrast, exposure to DE-71 resulted in an increase in light-seeking behavior, as indicated by the phototaxis test results of zebrafish larvae [63].

**Table 3:** Comparative analysis of variations in the practical application of phototaxis behavior response.

Compounds	Stage	Experiment Duration		Behavior Evaluation Parameters	Ref.
		Duration in the Dark Chamber	Duration after Removing the Partition		
TCC	144 hpf	10 min	5 min	Number of larvae swimming from the dark chamber into the illuminated chamber.	[62]
BDE-99	120 hpf	5 min	5 min, 10 min		[16]
Phe	7 dpf	2 min	30 min		[46]
Boscalid	8 dpf	10 min	1 min		[17]
TPhP	144 hpf	5 min	1 min		[44]
DE-71	15 dpf	2 min	1 min		[63]
BPS	5 dpf	15 min	15 min	Numbers of larvae travelled to the light chamber within 1 min.	[70]

### 3.4. Light-Dark Preference Test

The light–dark preference test is a well-established approach to assessing anxiety-like behavior in mice and investigating the mechanisms underlying drug-induced neurobehavioral changes [71]. Recently, this test has also been recognized as an extremely valuable tool for evaluating the vision of zebrafish as well as behavioral alterations due to retinal damage and regeneration [38]. The light–dark preference test is particularly advantageous for studying the behavioral neuroscience of zebrafish, as opposed to rodents, who typically prefer darkness [72]. Research involving zebrafish commonly utilizes this test, including high-throughput analysis of neural phenotypes and drug screening [22].

Typically, the light–dark preference test involves exposing zebrafish to alternate light and dark cycles [73]. The experimental setup can vary in terms of the developmental stage of zebrafish larvae, the number of holes in the porous plate, and the duration of the experiment, including the duration of light and dark conditions, as summarized in Table 4. Several key behavioral parameters are measured during the light–dark preference test, including movement times, average speed, maximum speed, chemotaxis, and the total distance traveled [74].

Exposure of zebrafish to agrochemicals, specifically the phenylurea herbicides linuron and pyrethroid esfenvalerate, has been demonstrated to exert inhibitory effects on locomotor activity [75,76]. Notably, locomotion under dark conditions was also found to be reduced following exposure to 2,2′,4,4′-tetrabromodiphenyl ether (PBDE-47) [77]. In contrast, exposure to BPS, retinoic acid, and the substitute for perfluorooctane sulfonic acid, F-53B, results in hyperactive swimming behavior, as indicated by the results of light-dark preference tests [69,78,79]. Furthermore, the presence of pharmaceutical compounds in aquatic environments can exert an influence on the development of zebrafish eyes. For instance, exposure of zebrafish to amitriptyline (AMI), venlafaxine (VEN), and sertraline (SER) leads to significant alterations in their swimming distance under both dark and light conditions [80]. Additionally, exposure to cyclophosphamide (CP) markedly inhibits the swimming speed of zebrafish larvae [81]. Conversely, the locomotor activity of zebrafish larvae remains unaffected by exposure to the beta-blocker atenolol [82]. However, under prednisolone exposure, the light–dark preference test results reveal that prednisolone alters the embryonic response to darkness, although these changes are not directly mediated by visual alterations [49].

**Table 4:** Comparative analysis of variations in the practical application of light-dark preference test.

Compounds	Stage and Well Plate	Protocols	Behavior Evaluation Parameters	Ref.
Retinoic acid	120 hpf, 24 well plate (1 fish/well)	Adaptation to 10 min in the dark was followed by a 10 min light (visible light) interval followed by a 10 min dark (infrared light) interval and repeated for 50 min.	Net speed change (or difference) in average swimming speeds between the last minute of 1 light state and the first minute of another light state and the average swimming speed of 10 min intervals for each state (light or dark)	[78]
AMI, VEN, SER	144 hpf, 96-microwell plates	Adaptation to light for 10 min, 16 alternating dark and light periods, each 5 min long, 90 min.	Swimming distance	[80]
Prednisolone	120 hpf, the apparatus consisted of a 6 cm diameter petri dish marked with a grid floor (4.5 mm2; approx. one body length), equally divided into a white and black area. The floor of the dish was further divided into three circular areas (outer, middle and central).	Individual embryos were initially acclimated within a small transparent chamber positioned within the central area for 1 min to explore the tank for 5 min.	The percentage of time spent in each compartment (light vs. dark), the proportion of time within each of the three circular areas within the light compartment, and the total level of activity (total number of lines crossed) while in the white compartment.	[49]
PBDE-47	4, 5, and 6 dpf, 96-well microplates	Adaptation to the dark took 20 min, and then recording started with a dark period of 10 min, followed by three cycles of alternating 10 min light and dark periods. The total testing time was 70 min.	Distance moved	[77]
Atenolol	exposed to atenolol for 7 days, 96-well plates	Alternating 10 min periods of light and dark, beginning with a dark period (i.e., dark photokinesis), for a total of 50 min.		[82]
Linuron	7 dpf larvae, 96-well plate			[76]
Esfenvalerate	5 dpf, 6 dpf, or 7 dpf, 96-well plates	Alternating light-dark periods (10 min light–10 min dark–10 min light–1 min dark) for 50 min.		[75]
F-53B	120 hpf, 24-well plates (one larva for each well)	Adaptation was performed 30 min before the start of the test, followed by a light (10 min) –dark (10 min) –light (10 min) stimulus.	Swimming speed (mm/s)	[69]
CP	larvae, 48-well plates	Alternating light-dark periods (10 min light–10 min dark–10 min light–1 min dark) for 50 min.	Swimming speed (mm/min)	[81]
BPS	After 120 h of BPS exposure, 24-well plates (1 larvae/well, 2 mL solution/well)	Alternating light-dark periods (1 min light–1 min dark–1 min light–1 min dark) for 4 min.	The traveled distance per minute, 4 min total distances, and average speed.	[79]

### 3.5. Free Swimming

Swimming ability is a critical behavioral parameter for fish as it plays a pivotal role in their survival, reproduction, and defense [83]. Impairments in visual and sensory processes can lead to lower swimming capability in zebrafish, which can have a negative impact on feeding, growth, and predator avoidance, eventually jeopardizing their ability to flourish in natural settings [68]. The free-swimming activity test assesses the visual function of zebrafish by monitoring their movements in an unrestricted state. This test involves placing zebrafish in a transparent tank, observing their swimming behavior without any interference from shock or noise, and recording their swimming trajectory under continuous visible light using a high-resolution camera. One of the primary parameters assessed in this test is swimming speed [68]. The specific protocols for conducting free swimming tests can vary, including the developmental stage of zebrafish larvae and the experimental design. More detailed information can be found in Table 5.

Nanoplastics (NPs) are emerging contaminants that have adverse effects on the environment and can serve as carriers for other coexisting pollutants [84,85]. For instance, the sunscreen butyl methoxydibenzoylmethane (Avobenzone, AVO) can adsorb onto the surface of nanoplastic particles, hindering their degradation and preventing their entry into the aquatic environment via conventional wastewater treatment methods 52. Exposure to NPs has been found to significantly decrease the swimming activity of zebrafish larvae. Further, zebrafish larvae showed a significant reduction in swimming activity when exposed to the combined exposure of NPs and AVO [86,87]. Nevertheless, research has demonstrated that retinoic acid at a dose of 2 nM improves the free swimming speed of zebrafish under both light and dark conditions [78].

**Table 5:** Comparative analysis of variations in the practical application of free swimming.

Compounds	Stage and Well Plate	Experimental Plan	Behavior Evaluation Parameters	Ref.
Retinoic acid	120 hpf, 24-well plates (1 fish/well)	Two 20 min swim tests in the light and dark	Swimming speed (mm/s)	[78]
The combined exposure of NPs and AVO	Exposure lasted for 144 h of morphologically normal zebrafish larvae, 24-well plates (1 larva/well)	After 10 min of daptation, a 15 min swim test was performed under continuous visible light.		[87]
	120 hpf, 24-well plate (1 larva/well)			[86]

### 3.6. Visual Avoidance Behavior

Zebrafish embryos exhibit unique avoidance behavior, which makes them a valuable model for assessing their motion detection ability. Simple animations composed of basic shapes are employed to elicit avoidance responses in zebrafish during the early developmental stages. The perception of motion necessitates image formation and tracking of positional changes over time, encompassing intricate visual processes that interact with the nervous system [49]. The specific implementation of this test varies depending on the growth stage of the zebrafish, the patterns employed for visual stimulation, and the individual experiment plan, as outlined in Table 6.

Previous research has demonstrated that zebrafish larvae exposed to MPs, Cu, prednisolone, PCB-95, and combined exposures of Cu and MPs exhibit abnormal behavior during early developmental stages. These abnormalities manifest as either a lack of significant avoidance response towards visual stimuli or reduced avoidance behavior [49,88,89].

**Table 6:** Comparative analysis of variations in practical applications of visual avoidance behavior.

Compounds	Stage and Well Plate	Visual Stimuli Patterns	Protocols	Behavior Evaluation Parameters	Ref.
Prednisolone	144 hpf, six-well plates	Produced by Microsoft PowerPoint, it consists of two black rectangles (0.4–14.6 cm) placed parallel to each other.	Acclimatization was performed on a white screen for 5 min, followed by 5 min of recording before stimulus onset, after which the animation was played for 5 min.	(Pre-animation, during, and post-animation) The amount of time embryos spent in the outer area of the well	[49]
PCB-95	5 dpf, 6-well plates	Produced by Microsoft PowerPoint, red lines (RGB values were 255, 0, 0) were spaced 1 mm apart and moved from the top to the bottom relative to the dish at a speed of 7 mm every 8 s.	Exposure to a uniform white background without visual stimuli was performed for 10 min, followed by moving the red line for 10 min and then returning to the white background for 10 min.	Swim speed, percentage of larval edge preference, and percentage of larval avoidance	[88]
MPs, Cu, and combined exposures of Cu and MPs	14 dpf, 6-well plates	Produced by Microsoft PowerPoint, static or bouncing two-dimensional red discs (1.35 cm diameter, RGB 255, 0, 0)	Alternating 10 min of a white background and 10 min of a red bouncing ball appeared in the upper half of the well and moved from left-right-left on a 2 cm straight trajectory at 1 cm/s	The time spent in the lower half of the well	[89]

## 4. Mechanism of Ocular Toxicity

### 4.1. Oxidative Stress and Inflammation

**Figure 4:**
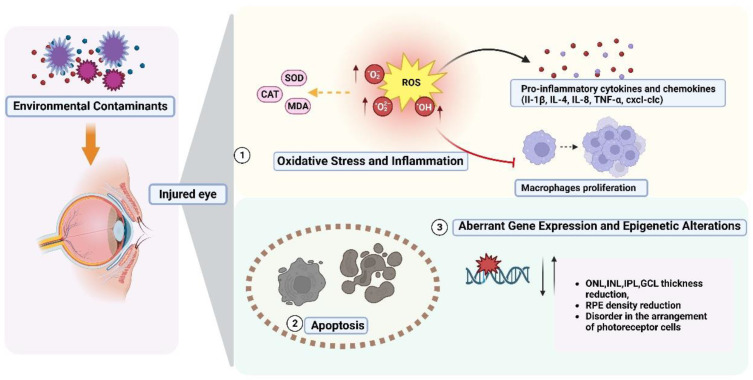
Potential mechanisms of eye damage from environmental contaminants in zebrafish.

Environmental contaminants have the potential to impact the ocular immune system by inducing oxidative stress, resulting in abnormal production of cytokines and chemokines and altered immune cell function (Figure 4). Oxidative stress is a major cause of photoreceptor damage, and it can be directly caused by the accumulation of reactive oxygen species (ROS) and aberrant metabolic activities [90]. To counteract oxidative damage and maintain cellular redox homeostasis, the antioxidant defense system relies on a combination of antioxidant enzymes and non-enzymatic antioxidant molecules. Superoxide dismutase (SOD) and catalase (CAT) are key antioxidant enzymes that act as the first line of defense against oxidative toxicity, and elevated levels of malondialdehyde (MDA), a consequence of lipid peroxidation, serve as indicators of oxidative damage [91,92]. Previous studies have shown that environmental contaminants, including endocrine disruptors (e.g., TCC [93], TPhP [94]), antidepressants [91,95], the agricultural chemical boscalid [96], and MPs [97,98], induce oxidative stress in ocular tissues, leading to structural damage and dysfunction of ocular cells.

Oxidative stress also contributes to the production of pro-inflammatory cytokines, which may negatively impact the immune system. Cytokines and chemokines play critical roles in mediating the innate immune response. Pro-inflammatory cytokines such as IL-1β, IL-4, IL-8, TNF-α, and cxcl-clc are recognized as key regulators of the inflammatory process and serve as significant markers during the innate immune response [99]. Furthermore, changes in immune cells have a significant impact on the immunological response. Macrophages in zebrafish act as phagocytes and have an intrinsic immunological role in battling infectious pathogens [100]. Zebrafish exposed to TCC alone or co-exposed to MPs and F-53B exhibited increased transcript levels of pro-inflammatory cytokines and chemokines [99,101]. Notably, TCC directly inhibits macrophage proliferation, thereby reducing the resistance of ocular tissues to infection and increasing the likelihood of lesions [93,99].

### 4.2. Apoptosis

Apoptosis represents a prevalent mechanism underlying retinal ganglion and photoreceptor cell damage [56]. Environmental contaminants, including TPhP [94], F-53B [69], BPS [56], PCB1254 [102], Phe [46], crude oil [61], and BDE99 [16], have been demonstrated to induce cell apoptosis. Specifically, TPhP [94], F-53B [69], and BPS [56] have been shown to enhance cell apoptosis in the ocular region of zebrafish, ultimately causing visual impairment via the mitochondria-mediated cell death pathway.

### 4.3. Aberrant Gene Expression and Epigenetic Alterations

Multiple environmental contaminants have been demonstrated to have an impact on the structure and morphology of the retina, causing morphological defects and changes in the thickness of different retinal layers. These changes include disruptions in the arrangement of photoreceptor cells in the ONL of the retina [56], as well as decreases or increases in thickness and the loss of retinal ganglion cells [46]. Consequently, these changes can lead to a decrease or impairment in vision [16,69,92,103]. These alterations are likely attributed to abnormal gene expression, which affects the functionality and stability of ocular cells, consequently causing damage to ocular tissues. Previous studies have suggested that a decrease in ONL thickness may be associated with the down-regulation of the optin gene in optic cones [62]. Furthermore, the down-regulation of genes crucial for controlling eye morphology and retinal development can impact the structure and development of the eye and retina, ultimately resulting in the occurrence of ocular malformations [104].

Previous investigations have provided evidence indicating the influence of a diverse range of environmental contaminants on the genetic expression pertinent to retinal development and phototransduction in zebrafish. These contaminants encompass a range of EDCs such as PBDE-47 [77], DE-71 [63,103], BDE99 [16], Phe 6, crude oil [61], PCB1254 [15], BPS [56], TCC [62,105], and F-53B [69]. Additionally, the brominated flame retardant TPhP [44,94], the agricultural chemical boscalid [17], and PPCPs such as amitriptyline [80], venlafaxine [80], sertraline [80], and AVO [86,87], as well as microplastics [86,87,106] have demonstrated their ability to modulate the expression of genes closely associated with visual development in zebrafish. Consequently, these alterations in gene expression have been observed to induce notable changes in the visual behavior of fish. Table 7 summarizes the effects of the abovementioned environmental contaminants on genes related to visual development.

## 5. Challenges and Prospects

Zebrafish serve as a valuable model organism for investigating visual systems due to their resemblance to other vertebrates in terms of their visual system [21]. The assessment of zebrafish visual behavior through quantitative analysis provides insights into their response to visual stimuli, allowing for noninvasive detection of potential eye lesions, identification of toxic effects on vision, and rapid evaluation of the impacts of pollutants on the eye and visual system. However, the behavioral response does possess certain challenges in the evaluation of ocular toxicity in zebrafish. These techniques are unable to directly elucidate the underlying cellular and molecular processes, and there is a lack of standardized protocols for behavioral testing. Additionally, zebrafish models cannot entirely replace mammalian models. Therefore, it is crucial to consider and address these challenges fully when using zebrafish models for behavioral assessment of ocular toxicity.

(1) The direct correlation between ocular toxicity and biological tissue levels, including genes, proteins, tissues, and physiology, cannot be readily determined when using behavioral responses to assess the potential toxicity of environmental contaminants [32]. Therefore, further quantitative investigations employing alternative methodologies are required to establish whether the observed damage to the visual system is directly attributed to the pollutants under scrutiny. In vision science research, the up-regulated or down-regulated trend of key visual pathways can be verified through molecular detection of gene and protein expression related to retinal development and function. Additionally, histological analysis using microscopy and physiological analysis through electroretinograms can serve to corroborate these trends. Further research is necessary to determine whether environmental contaminants have genetic effects on visual system toxicity in zebrafish;
(2) Although many visually mediated behavior testing methods have been applied to zebrafish research, there is currently a lack of unified standards for each method in actual application. Therefore, it is crucial to establish zebrafish behavioral standards under the guidance of vision. For example, in behavioral experiments, it is essential to standardize key experimental parameters such as light intensity, pollutant co-incubation time, and recording time to improve the repeatability of research results across different teams and strengthen the persuasiveness of conclusions based on zebrafish behavioral data. This will promote the construction of zebrafish behavior test standards;
(3) Considering the different routes of environmental pollution entry into zebrafish and mammalian visual systems is important when utilizing zebrafish as a predictive model. There is no doubt that environmental pollutants exist in a variety of environmental substrates, including water, sediments, soil, air, and biota. As a result, both zebrafish and human eyes are inevitably exposed to environmental pollutants, which can cause ocular damage [11,124,125,126]. This commonality indicates the potential shared effects of environmental pollutants on the visual system across different species. Researchers can take advantage of the benefits of zebrafish for rapid evaluation, drug screening, and preliminary studies of mechanisms. Nevertheless, multiple animal models, multiple tissue levels, and multi-angle studies are necessary to further clarify the mechanism of ocular toxicity caused by environmental contaminants.

## 6. Conclusions

Environmental contaminants have recently been the subject of increasing attention due to their potential influence on the visual system, which is a vital sensory organ for the survival, development, and learning activities of organisms. However, the currently used model organisms such as mice limit their usefulness for ocular toxic applications due to their rod-shaped vision, slow development of the visual system, and greater reliance on smell, touch, and hearing. In contrast, zebrafish, which possess cone vision similar to humans, have presented unprecedented advancements in visual system toxicity research. Observing physiological and behavioral changes in zebrafish enables the swift assessment of potential ocular toxicity caused by pollutants, revealing different levels of damage. Future research combining standard visually mediated behaviors with comprehensive quantitative investigations promises new insights into the mechanisms underlying the ocular toxicity of environmental contaminants.

## Figures and Tables

**Table 7:** The effects of the abovementioned environmental contaminants on genes related to visual development.

Categories	Compounds	Water Solubility	Exposure Dose	Exposure Time	Changes in Genes Associated with Visual Development	Ref.
EDCs	PBDE-47	Insoluble in water	500 μg/L	/	*rx2*, *opn1sw1*, *opn1mw1*, *opn1sw2* ↓; *opn1lw*1 ↑	[107,108]
	DE-71	water-soluble	0.32, 3.58, and 31.0 μg/L	from 2 hpf to 15 dpf	*zfrho*, *zfgr1* ↑ (3.58, and 31.0 μg/L)	[63]
	BDE-99	9e-10 mg/mL	5, 50 μg/L	from 3 hpf to 120 hpf	*opn1sw1*, *opn1sw2*, *opn1mw1*, *opn1mw2*, *opn1lw2*, *rho* ↓	[16,109]
	Phe	1.10 mg/L	3.56, 35.6 and 356 μg/L	from 2 hpf to 72 hpf	*Zeb1* ↓, *Mitf* ↑	[46,110]
	Crude oil	6.6e-3 mg/L	12.8, 38.5, 64.2, and 89.8 μg/L	from 4 hpf to 72 hpf	*arr3b*, *gnat2*, *opn1mw*, *rgr*, *rho*, *rpe65a*, *sws1* ↓ (38.5, 64.2 and 89.8 μg/L); *crx*, *pde6c*, *pde6h* ↓ (64.2, 89.8 μg/L)	[62,111]
	PCB1254	extremely low	0.125, 0.25, 0.5 and 1 mg/L	7 days of continuous exposure	*SWS2* ↓ (0.125,0.25,0.5 and 1 mg/L); *CRX*, *RHO*, *SWS1* ↓ (0.5, 1 mg/L)	[15,112]
	BPS	Insoluble in water	1, 10, 100 and 1000 μg/L	from 2 hpf to 120 dpf	*zfrho* ↑ (1,10 μg/L); *zfblue* ↑ (1000 μg/L); *zfred*, *zfgr1* ↑ (1, 10, 100, 1000 μg/L)	[57,113]
			4400 nM	from 2 hpf to 24 hpf	*opn1sw1*, *arr3a*, *pde6h*, *gk1b* ↑, *opn1mw2*, *opn1lw1*, *opn1lw2*, *rho*, *pde6a* ↓ (4 nM); *opn1sw1*, *opn1sw2*, *opn1mw1*, *opn1mw2*, *opn1mw3*, *opn1lw1*, *opn1lw2*, *arr3a*, *pde6h*, *gk1b* ↓, *rho*, *pde6a* ↑ (400 nM)	[79]
	TCC	0.11 mg/L	0.20, 40.0 µg/L	from 2 hpf to 6 dpf	*opn1sw2*, *opn1mw1*, *opn1mw3*, *opn1lw1*, *opn1lw2*, *rho* ↓	[62,114]
	F-53B	200 mg/L	0.15, 1.5 and 15 μg/L	from 2 hpf to 120 hpf	*aldh1a2*, *cryaa*, *crybb*, *crygn*, *mipa*, *pax6*, *rx1*, *gant1*, *rho*, *opn1sw*, *opn1lw* ↓	[69,115]
BFRs	TPhP	Insoluble in water	4, 100 μg/L	from 2 hpf to 144 hpf	*zfrho*, *zfred*, *zfgr1*, *zfuv*, *zfblue*, *rhodopsin* ↓ (100 μg/L); *retinoschisin 1a* ↓ (4 μg/L)	[27,116]
			0.1, 1, 10, and 30 μg/L	from 2 to 144 hpf	*zfrho*, *opn1sw1*, *opn1sw2*, *opn1mw1*, *opn1mw2*, *opn1mw3*, *opn1mw4*, *opn1lw1*, *opn1lw2 ↓*	[44]
Agricultural chemicals	Boscalid	4.6 mg/L	0.3, 0.6, and 1.2 mg/L	from 2 hpf to 4 dpe	*opn1sw1*, *opn1mw1*, *opn4.1*, *rho* ↑ (0.3 mg/L); *opn1sw1*, *opn1mw1*, *opn1lw2*, *opn4xb*, *opn4.1*, *rho*, *rhol* ↓ (1.2 mg/L)	[17,117]
				from 2 hpf to 8 dpe	*opn1sw1*, *opn1lw2*, *opn4xb*, *rho* ↑ (0.3 mg/L)	[17]
PPCPs	AMI	9.71 mg/L	0.3, 30 μg/L	to 144 hpf	*pax 6* ↓ (30 μg/L)	[80,118]
	VEN	267 mg/L			*pax 6*, *otx2* ↓	[80,119]
	SER	3.8 mg/mL	0.1, 10 μg/L		*pax 6* ↓, *otx2* ↑	[80,120]
	AVO	2.2 mg/L	10 μg/L	from 2 hpf to 144 hpf	six6 ↓; *lhx9*, *pax2*, *pax6* ↑	[87,121]
MPs	NPs	Insoluble in water			*lhx9*, *six6* ↓; *six3*, *pax2*, *pax6* ↑	[87,122]
	MPs	Insoluble in water	1 mg/L	from 3 hpf to 1120 hpf	*Zfrho* ↑	[108,123]

## Data Availability

Not applicable.

## Abbreviations

BDE99	2,2’,4,4’,5-pentabromodiphenyl ether
PBDE-47	2,2′,4,4′-tetrabromodiphenyl ether
AMI	Amitriptyline
AVO	Avobenzone
BPS	Bisphenol S
BFRs	Brominated flame retardants
CAT	Catalase
CP	Cyclophosphamide
EDCs	Endocrine disrupting chemicals
GCL	Ganglion cell layer
hpf	Hours post-fertilization
INL	Inner nuclear layer
IPL	Inner plexiform layer
MDA	Malondialdehyde
MPs	Microplastics
NPs	Nanoplastics
OKR	Optokinetic reflex
OMR	Optomotor response
ONL	Outer nuclear layer
OPL	Outer plexiform layer
PPCPs	Pharmaceutical and personal care products
Phe	Phenanthrene polycyclic aromatic hydrocarbon
PBDE	Polybrominated diphenyl ether
PAHs	Polycyclic aromatic hydrocarbons
ROS	Reactive oxygen species
RGC	Retinal ganglion cells
RPE	Retinal pigment epithelium
SER	Sertraline
SOD	Superoxide dismutase
TCC	Triclocarban
TPhP	Triphenyl phosphate
UV	Ultraviolet
VEN	Venlafaxine
